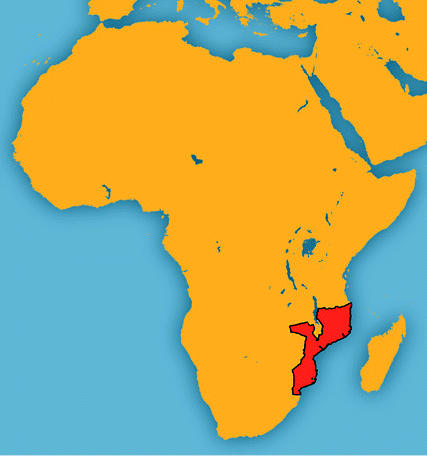# The Beat

**Published:** 2005-01

**Authors:** Erin E. Dooley

## Smoky Horror Picture Show?

At the American Medical Association’s 2004 annual meeting, the group’s policy-making House of Delegates adopted a resolution urging the film industry to give an “R” rating to movies with scenes of characters smoking. The goal of the resolution is to reduce the amount of smoking seen in movies, and to limit those scenes to movies seen only by adults. Speaking before the assemblage, Stephen Hansen, coordinator of the association’s Tobacco Control Coalition, cited several recent studies showing that the number of smoking scenes onscreen is up from an average of 5 scenes per hour in the 1950s to 11 today. Other studies suggest that film depictions of smoking may correlate with the onset of smoking in youth.

## EU Bans Phthalates in Toys

In September 2004 the European Competitiveness Council voted to ban three phthalates from all products intended for children and to prohibit the use of three others specifically in toys and other items intended to be chewed or sucked by very young children. These chemicals, which are used to soften vinyl plastic, have been linked with reproductive and liver effects, and are known to leach from products that contain them. More than 900 tons of phthalates are produced each year.

Once the measure has been adopted formally by the council it will be sent to the European Parliament for a second reading. The European Commission will be charged with overseeing the implementation of the ban.

## Roaming Foam May Find a Home

The polystyrene foam that helps boat docks stay afloat can break off in large chunks, littering the lakescape and posing a boating hazard. Foam is traditionally very hard to recycle because it is wet and oily, and often contains metal screws and other items that can damage recycling machines. Now the Missouri-based company BioSpan Technologies has developed a solvent that dissolves the wet, dirty chunks at a ratio of more than 3 cubic yards of foam per gallon of solvent. The dissolved blend is then mixed with recycled asphalt to patch potholes. Other products made with the blend are used to preserve cement, wood, and metal.

## Coco Locomotion

Coconuts are the latest plant to be tapped for bio-based fuels. In October 2004, a unit of the Philippine National Oil Company opened the first cocodiesel plant. The plant is meant to show Filipino farmers how the technology can benefit them and their communities. Coconut oil and methanol are the major raw materials used to produce a biodiesel that burns cleaner than regular diesel without the need for engine modifications. The fuel costs about 8¢ less per kilometer to use, and the process also yields glycerine, which can be used to make soap. Some Filipino government vehicles are already using a 1% blend of cocodiesel as part of a presidential drive to reduce vehicular pollution.

## Targeting Mosquitoes Online

Ever wonder whether those swarming mosquitoes in your backyard are carrying West Nile virus or some other disease? Researchers at Texas A&M University are developing a web-based real-time system that researchers and the public will be able to use to see where disease-carrying vectors have been spotted. The Mosquito Spatial Information Management System will map disease occurrence, epidemiology, and control procedures. Jim Olson, an entomologist on the team, said the system is just a small part of a larger multiagency project to determine the level of mosquito resistance to pesticides. This information will help pest management officials choose the most appropriate mosquito control measures for any given locality.

## Mozambique Phases Out Leaded Gas

In August 2004 Mozambique announced its intention to ban the importation of leaded gasoline by the end of the year. The decision followed government approval of an action plan by the Leaded Gasoline Phase-out Task Force, a multiagency group working to facilitate the replacement of leaded gas with safer options, and to educate the public on the health and societal benefits of doing so. The task force plans to completely phase out the use of leaded gasoline in the country by mid-2005.

Most lead exposure is to airborne lead and lead in dust and soil. Excessive lead exposure is associated with cognitive impairment, stunted growth, and permanent brain damage and mental retardation. Lead has been found in vegetables grown in urban African gardens at levels higher than U.S. EPA allowable limits.

## Figures and Tables

**Figure:**
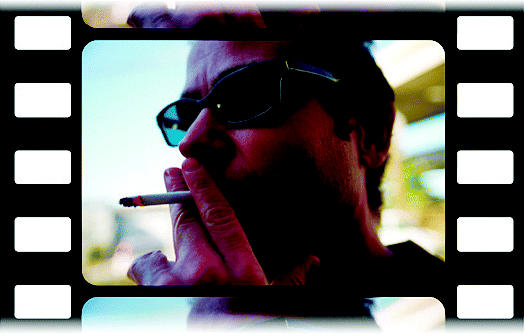


**Figure:**
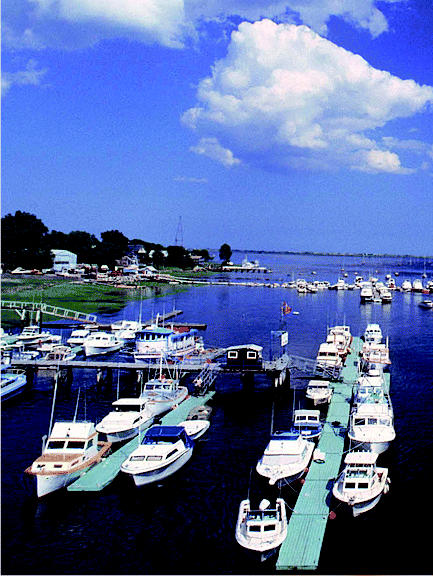


**Figure:**
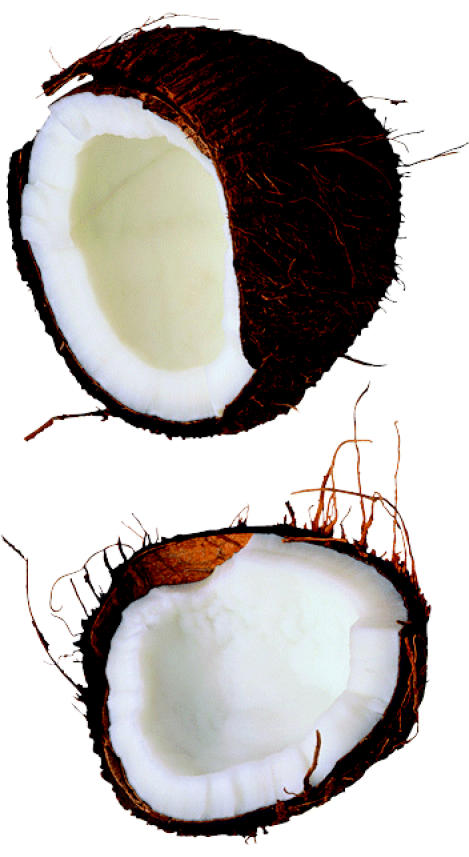


**Figure:**